# The global trends and distribution in tumor-infiltrating lymphocytes over the past 49 years: bibliometric and visualized analysis

**DOI:** 10.3389/fimmu.2024.1511866

**Published:** 2025-01-06

**Authors:** Beibei Wu, Ding Luo, Xuejie Wang, Chen Qiao, Rui Li, Jian Liu

**Affiliations:** ^1^ Beijing Traditional Chinese Medicine Office for Cancer Prevention and Control, Xiyuan Hospital, China Academy of Chinese Medical Science, Beijing, China; ^2^ Department of Information, Xiyuan Hospital, China Academy of Chinese Medical Science, Beijing, China; ^3^ Department of Oncology, Xiyuan Hospital, China Academy of Chinese Medical Science, Beijing, China

**Keywords:** tumor-infiltrating lymphocytes, adoptive cell therapy, predictive biomarker, PD-L1, bibliometrics

## Abstract

**Background:**

The body of research on tumor-infiltrating lymphocytes (TILs) is expanding rapidly; yet, a comprehensive analysis of related publications has been notably absent.

**Objective:**

This study utilizes bibliometric methodologies to identify emerging research hotspots and to map the distribution of tumor-infiltrating lymphocyte research.

**Methods:**

Literature from the Web of Science database was analyzed and visualized using VOSviewer, CiteSpace, Scimago Graphica, R-bibliometrix, and R packages.

**Results:**

Research on tumor-infiltrating lymphocytes began in 1975 and has experienced significant growth, particularly after 2015. Leading contributors include the United States, the National Cancer Institute, the journal Cancer Immunology Immunotherapy, and researcher Steven A. Rosenberg. Other prominent contributors include China, the National Institutes of Health, researcher Roberto Salgado, and the Journal of Immunology. Prominent institutions in the USA and Europe occupy central roles within collaborative networks. Financial support plays a pivotal role in driving research advancements. Keyword clustering analysis reveals four primary knowledge domains: adoptive cell therapy; the prognostic value of TILs; PD-1/PD-L1 and TILs; and prognostic studies of TILs across various cancers. Keyword and reference analyses further indicate that “adoptive cell therapy,” “the prognostic value of TILs,” and “immune checkpoint inhibitors and TILs” are central themes in current and future research. Combination therapies; tumor neoantigens; gene editing; dominant population selection of TILs therapy; TILs in Tumor microenvironment; emerging predictive biomarkers; TILs in predicting the efficacy of neoadjuvant chemotherapy and immunotherapy; the relationship between TILs and PD-L1; TIL-based patient stratification; tertiary lymphoid structures; and TIL evaluation through digital pathology and artificial intelligence are identified as key areas of interest.

**Conclusions:**

This analysis highlights the increasing academic focus on tumor-infiltrating lymphocyte research and identifies key recent themes in the field such as prognostic value of TILs, personalized treatments, and combination therapies.

## 1 Introduction

The tumor microenvironment (TME) plays a pivotal role in tumor growth and progression (1). As a crucial component of the TME, tumor-infiltrating lymphocytes (TILs)—including T cells, B cells, and NK cells—are essential for the anti-tumor immune response (2). Recently, the widespread application of immunotherapy in cancer treatment has significantly increased interest in TILs research. Autologous TILs therapy has demonstrated remarkable efficacy and safety, particularly in melanoma (3, 4). This treatment involves the ex vivo expansion of a patient’s TILs followed by their reinfusion, which can induce significant anti-tumor responses. Advancements in technology, particularly in single-cell sequencing, immunohistochemistry, and multiplex fluorescence microscopy, enable researchers to more accurately identify and analyze the types, functions, and dynamic changes of TILs (5–7). Moreover, much research has established that the quantity and functional state of TILs is closely associated with the prognosis of cancers (8–10). For example, high densities of TILs, such as CD8+ T cells, are generally associated with better prognoses in multiple tumors, including melanoma, breast cancer, and liver cancer (7, 11, 12). Concurrently, the heterogeneity of TILs has garnered increasing attention, providing a critical basis for personalized treatment (7, 13, 14). Additionally, the importance of digital pathology (15–20), combination therapies (21, 22), and multi-marker joint assessments (23–26) is also increasing. In summary, TILs research is rapidly evolving and diversifying, offering essential foundations for understanding tumor immune escape mechanisms and developing novel immunotherapeutic strategies.

Bibliometric analysis employs statistical methods to elucidate the results and impact of scholarly research (27), facilitating scholars’ understanding of a discipline’s background, historical processes, fundamental knowledge, current status, developmental directions, and prospects. It also assesses collaborative networks among researchers and is crucial in formulating research policies, guiding journal selection, and facilitating academic evaluations (27–30). This approach offers a valuable, timely, replicable, and adaptable methodology (31).

TILs have become a focus in contemporary cancer research, with numerous novel and significant directions emerging. Correspondingly, the volume of literature is rapidly expanding, necessitating alignment with emerging trends and critical shifts in collective knowledge development. Therefore, this study utilizes bibliometric analysis to delineate the historical trajectory of TILs research, unveil global collaborative networks, and identify research hotspots. This approach permits a more quantifiable, objective, and comprehensive analysis of the field. To our knowledge, this represents the first bibliometric analysis conducted on TILs research to address the following inquiries:

What is the overarching trajectory of research concerning TILs?

Which nations, institutions, journals, authors, and funders exert the most significant influence within this research purview?

In which primary disease areas are TILs most commonly applied?

What are the pivotal research themes within this domain? Which studies are regarded as seminal milestones? What are the current research hotspots? What are the potential future research directions?

## 2 Materials and methods

### 2.1 Data sources and collection

This study utilized the Web of Science as its database due to its high-quality academic literature and robust data support. It’s widely used by researchers for bibliometric analysis. To mitigate potential errors from database updates, we acquired all source data from the WOS Core Collection within a single day (May 10th, 2024). Given concerns about redundancy and peripheral literature with “topic” retrieval, and after reviewing the MeSH database, our study opted for “title” retrieval of “tumor-infiltrating lymphocytes”. Our data collection period spanned from January 01, 1965, to April 30, 2024, and only includes “article” and “review” document types. Language restrictions were not applied. The file format was confined to “Plain text”, and record content was specified as “Full Record and Cited References”. Additionally, to enable comparative analysis across different countries and institutions in the TILs field, we exported funding data and literature datasets categorized by the top countries and institutions of publication volume, all on the same day.

### 2.2 Bibliometric analysis and visualization

Microsoft Excel is used to identify annual publications and pivotal countries/regions, institutions, authors, journals, funding sources, references, and keywords. CiteSpace is designed to identify emerging trends and abrupt changes in scientific literature and is employed for co-occurrence networks and burst analysis of institutions, authors, keywords and references, and network layer overlay of journals. VOSviewer is used to visually represent co-occurrence networks of countries, journals, and keywords, as well as co-citation networks of journals. R-bibliometrix and R-studio are used to obtain detailed publication trends and influence indexes for institutions, authors, and journals. Scimago Graphica is used to generate the geographical visualization of national publications.

Additional details are available in **Supplementary Material 1**.

## 3 Results

### 3.1 Global publishing trend

**Figure 1:**
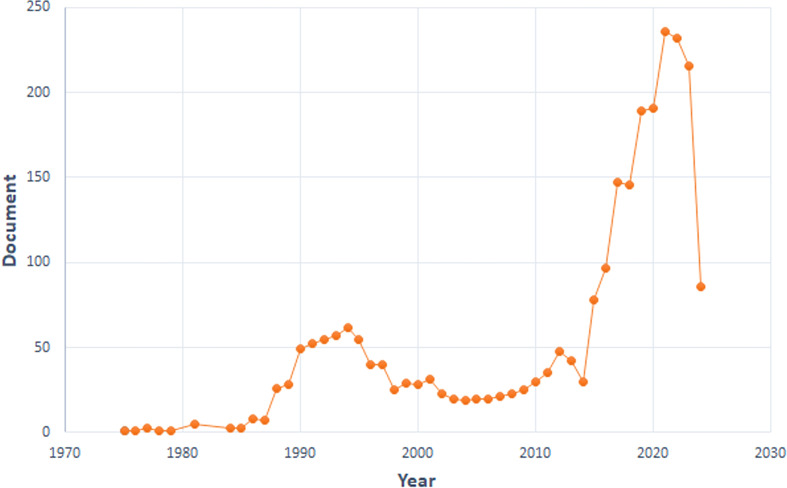
Annual trends of global publications.

We curated a dataset comprising 2,420 articles and 164 reviews from 1975 to 2024. We divided the research into three phases. Minimal development occurred from 1975 to 1987. The second phase (1988-2014) demonstrated steady growth. A pivotal shift occurred in 2015, marked by a significant surge in published papers. This upward trend persisted, reaching its peak in 2021 with 236 papers published (**Figure 1**), indicating that TILs research has entered a rapid development stage.

### 3.2 Global contribution in TILs research

#### 3.2.1 Co-countries analysis

**Figure 2:**
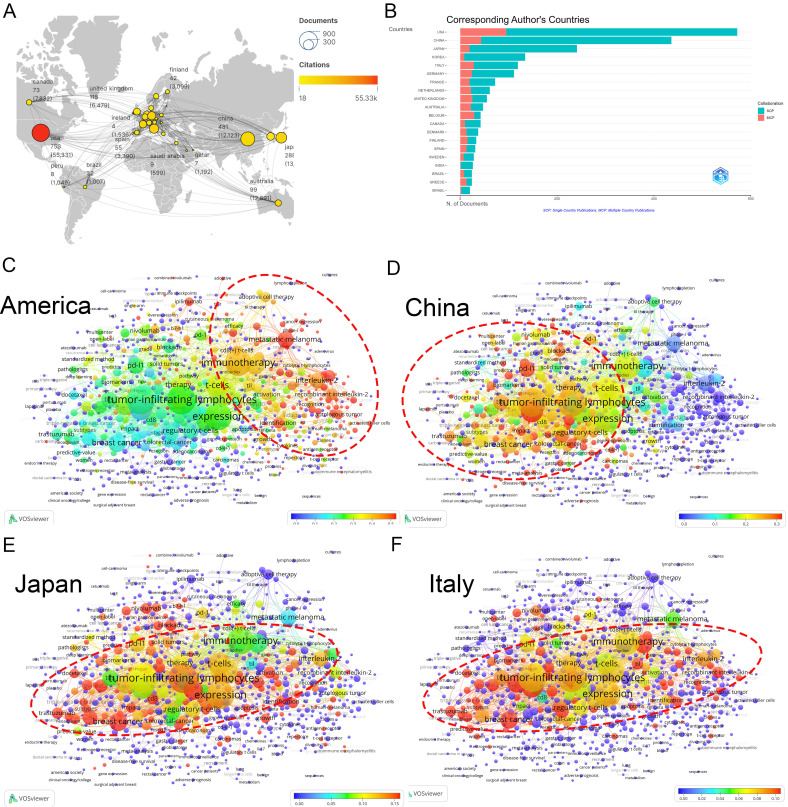
Analysis of countries. **(A)** A world map displaying the total publications from the top 30 countries/regions. The circle size represents the volume of publications, while the circle color indicates the volume of citations. The line thickness denotes the strength of collaboration. **(B)** Number of documents from corresponding authors’ countries. SCP: Single Country Publications, MCP: Multiple Country Publications. **(C)** Term contributions of the USA in TILs research. Each node represents a term, with the color denoting the frequency in articles from the USA relative to its overall frequency. The closer the color is to red, the higher the proportion. The larger and redder the node, the more prominent the role of the USA in global research pertaining to that term. **(D)** Term contributions of China in TILs research. **(E)** Term contributions of Japan in TILs research. **(F)** Term contributions of Italy in TILs research.

A total of 78 nations actively contribute to TILs research. The USA dominates with the highest number of publications (753), citations (55,331), and total link strength (519) (**Figures 2A, B**). Other key contributors include China (481), and Japan (288). Although South Korea ranked sixth in publications (145), it placed twelfth in citations (5,565), likely due to its limited collaboration (total link strength=70). The proportion of international co-authorships reflects relatively few joint publications (18.61%). In international collaborations, USA-centric partnerships occupied nine of the top 15 positions, predominantly with China (55 publications) (**Supplementary Material 2**).

An examination of countries’ keywords highlights the diverse research emphases across different nations (refer to “3.3 Analysis of keywords” for further details). The USA has significantly advanced various research areas within TILs, particularly focusing on adoptive cell therapy (**Figure 2C**). Conversely, China’s research has concentrated on the therapeutic and prognostic significance of TILs, and PD-1/PD-L1 immune checkpoint inhibitors (**Figure 2D**), Japan and Italy have also made important contributions to this area (**Figures 2E, F**).

#### 3.2.2 Co-institutions analysis

**Figure 3:**
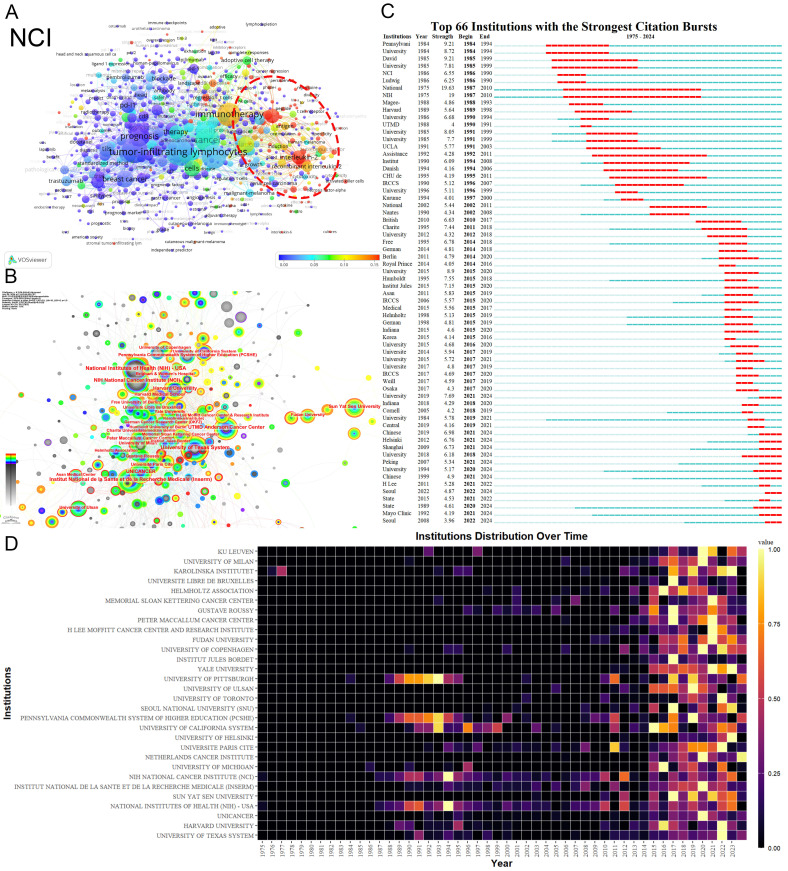
Analysis of Institutions. **(A)** Term contributions of the NCI in TILs research. **(B)** Institutional collaboration network. Each node represents an institution, with the node’s size reflecting the institution’s publication volume. Concentric circles around each node represent the number of publications by the institution in different years, with circle colors indicating various years. Wider circles denote higher publication volumes for the respective years. Lines connecting nodes represent collaborative relationships between institutions. The graph’s interpretation can be enhanced by considering both the size and color of the nodes. **(C)** The bursts analysis of institutional publication. “Strength” denotes the intensity of keyword bursts, while “year” indicates the initial appearance of the institution, as indicated by the dark blue band. “Begin” and “End” mark the start and end of the burst period, respectively, with the red band highlighting the duration of the burst. **(D)** Annual publication volumes for the top 30 institutions. The colors represent publication volume, with brighter colors indicating higher publication volume.

The National Cancer Institute(NCI) leads in publications(104) and citations (15,872) (**Tables 1**, **2**). **Figure 3A** shows that NCI focuses more on adoptive cell therapy. Furthermore, institutions such as the Jules Bordet Institute, the German Cancer Consortium, the National Institutes of Health, and Heidelberg University, despite having fewer publications, are distinguished by their high citations. Conversely, Sun Yat-sen University, the University of Ulsan, and Fudan University exhibit high publications but lower citations. Geographically proximate institutions show greater ease of collaboration. The center of the institutional collaboration network is characterized by numerous closely interconnected nodes, primarily comprising leading institutions in the USA and Europe. In contrast, Asian institutions, such as Sun Yat-sen University, Fudan University, the University of Ulsan, and Asan Medical Center, are comparatively isolated (**Figure 3B**). The burst analysis identifies several emerging institutions, including the University of Toronto and the University of Helsinki (**Figure 3C**). **Figure 3D** indicates the persistent contributors (NCI, University of Texas System, Harvard University, and the National Institutes of Health) and several recent contributors (UNICANCER, Sun Yat-sen University).

**Table 1:** The top 10 institutions contributing to publications on tumor-infiltrating lymphocytes.

ID	Organization	Documents	Citations	TLS	Country
1	NCI	104	15872	137	USA
2	Sun Yat-Sen University	57	1674	80	China
3	University of Texas MD Anderson Cancer Center	48	2558	128	USA
4	University of Ulsan	42	1204	89	South Korea
5	University of Pittsburgh	40	3280	111	USA
6	Karolinska Institutet	39	2077	153	Sweden
7	University of Texas	35	2271	17	USA
8	Fudan University	34	838	26	China
9	University of Milan	33	3761	226	Italy
10	Harvard University	30	1881	45	USA

TLS, In the co-occurrence network of institutions, the Total link strength attribute indicates the total strength of the co-authorship links of a given institution with other institutions.

**Table 2:** The top 10 institutions contributing to citations on tumor-infiltrating lymphocytes.

ID	Organization	Documents	Citations	TLS	Country
1	NCI	104	15872	137	USA
2	mem sloan kettering canc ctr	26	4468	122	USA
3	inst jules bordet	16	4250	142	Belgium
4	peter maccallum canc ctr	23	4137	199	Australia
5	charite	20	4046	180	Germany
6	University of Milan	33	3761	226	Italy
7	german canc consortium dktk	17	3695	160	Germany
8	the National Institutes of Health	13	3622	30	USA
9	University of Pittsburgh	40	3280	111	USA
10	heidelberg univ	10	2925	89	Germany

#### 3.2.3 Co-author analysis

**Figure 4:**
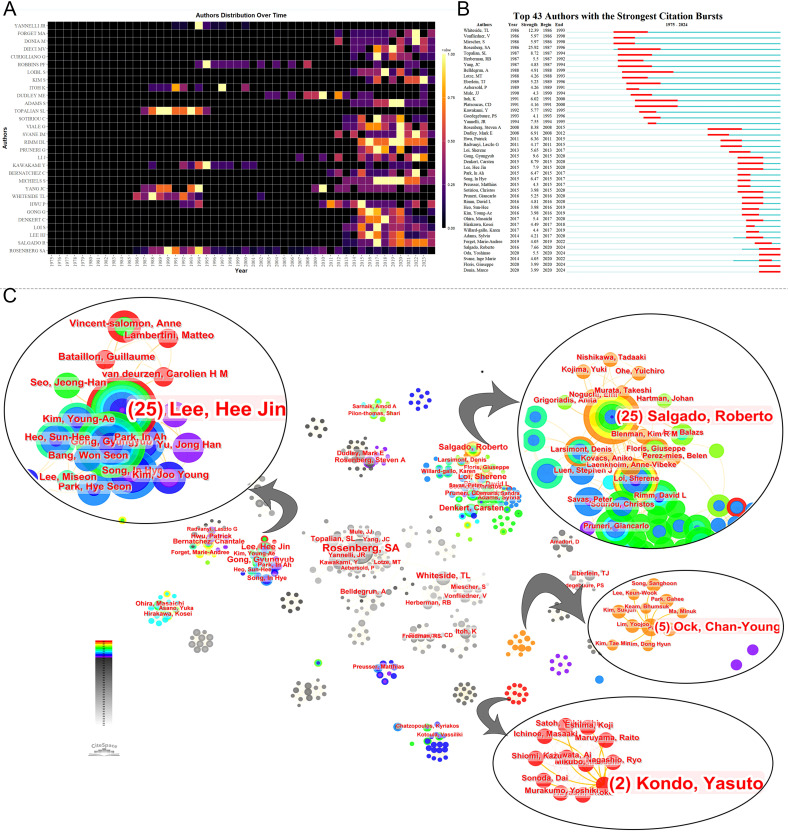
Analysis of authors. **(A)** Annual publication volumes for the top 30 authors. The colors represent publication volume, with brighter colors indicating higher publication volume. **(B)** The bursts analysis of authors’ publication. **(C)** Collaboration network of authors.

Prominent figures include Steven A. Rosenberg from the NCI, who leads in publications, local citations, total citations, h-index, and m-index (**Table 3**). His early contributions have been substantial (**Figures 4A, B**), especially in adoptive cell therapy and metastatic melanoma. Conversely, although Lee HJ, Whiteside TL, Hwu P, and Gong G are among the top ten publications, their local citations are comparatively lower (**Supplementary Material 3**). **Figure 4A** shows early contributors (e.g., Whiteside TL) and recent contributors (e.g., Lee HJ and Loi S). The bursts analysis reveals emerging scholars such as Roberto Salgado, Giuseppe Floris, Yoshinao Oda, and Marco Donia (**Figure 4B**). Roberto Salgado and Giuseppe Floris have close associations and substantial influence in breast cancer and TILs research (**Figure 4C**). Yoshinao Oda primarily investigates PD-L1 and TILs, whereas Marco Donia focuses on adoptive cell therapy. **Figure 4C** depicts the rise of numerous teams over time, including some emerging smaller teams, such as Ock Chan-Young’s, which are focusing on artificial intelligence and TIL evaluation.

**Table 3:** Top ten authors in a number of local citations.

Rank	Author	Documents	TC	LC	H_Index	M_Index	Institution	Country
1	Rosenberg SA	104	18344	2360	62	1.59	National Cancer Institute	USA
2	Loi S	34	9335	2118	26	2.167	University of Melbourne/Peter MacCallum Cancer Centre	Australia
3	Denkert C	32	9033	2020	26	2.364	Charité – Universitätsmedizin Berlin	Germany
4	Salgado R	41	8374	1917	24	2	University of Melbourne/Peter MacCallum Cancer Centre	Australia
5	Sotiriou C	20	7627	1790	18	1.5	Katholieke Universiteit Leuven	Belgium
6	Loibl S	18	6966	1656	15	1.364	German Breast Group (GBG)	Germany
7	Viale G	21	5801	1499	17	1.417	European Institute of Oncology	Italy
8	Adams S	18	5687	1405	15	1.364	New York University	USA
9	Demaria S	15	5506	1386	13	0.542	New York University School of Medicine	USA
10	Sirtaine N	6	5319	1304	6	0.5	Jules Bordet Institute	Belgium

TC, TotalCitations.

LC, LocalCitations, Local citations provide a more precise measure of an author’s influence within the field compared to total citation counts.

H-index, an academic’s h-index refers to the maximum number of *h* for which they have published *h* papers each cited at least *h* times. The h-index provides a relatively precise gauge of an individual’s scholarly accomplishments. A higher h-index signifies a greater influence on one’s publications.

M-index, by dividing the h-index by the number of years publishing, accounts for differences in professional longevity.

#### 3.2.4 Journals

**Figure 5:**
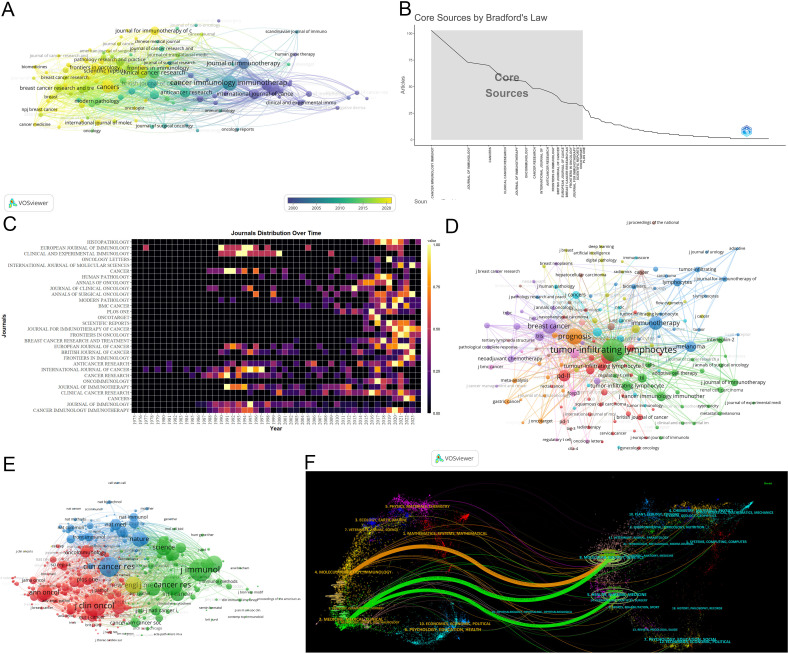
Analysis of journals. **(A)** Co-occurrence network of the journal. Nodes represent journals, with their size reflecting frequency. The timeline in the bottom right corner elucidates not the inaugural emergence of journals but rather their mean occurrence chronology. **(B)** Core Sources by Bradford’s Law. **(C)** Annual publication volumes for the top 30 journals. **(D)**Co-occurrence network of journal-keyword. Nodes represent either keywords or journals (identified by an uppercase “J” preceding the journal names). Links signify their correlation within the co-occurrence network, facilitating the exploration of thematic domains covered by the journals. The keywords in this figure consist solely of author keywords. **(E)** Map of co-cited journals. Each node represents a co-cited journal, node size corresponds to the number of co-citations, links indicate shared co-citations among journals, and colors indicate different clusters of co-cited journals. **(F)** The Overlay Maps of journals. On the left is the citing graph, and on the right is the cited graph. The curves represent citation links. In the left graph, the longer the horizontal axis of the ellipse, the more papers published in the journal; the longer the vertical axis, the more authors involved. A knowledge flow analysis revealed the evolutionary relationships between citing and cited journals.

*Cancer Immunology Immunotherapy* leads in publications(103) (**Figure 5A**). The *Journal of Immunology* leads in citations (7,856) and h-index (47) (**Table 4**) (**Supplementary Material 4**). According to Bradford’s Law, there are 18 core journals (**Figure 5B**). **Figure 5C** delineates early contributors (*Journal of Immunology, Cancer Research, International Journal of Cancer*), persistent contributors (*Cancer Immunology Immunotherapy*), and recent contributors (*Frontiers in Immunology, Cancers, Frontiers in Oncology, Journal for Immunotherapy of Cancer, Scientific Reports*). **Figure 5D** highlights the theme of journals, with recent contributor journals emphasizing PD-L1, prognosis, breast cancer, neoadjuvant chemotherapy, biomarkers, and adjuvant chemotherapy. Co-citation analysis identifies the *Journal of Immunology* (4,655) being the most frequently co-cited (**Figure 5E**). Overlay maps of journals reveal that those focusing on molecular biology and genetics are frequently cited by journals in molecular biology and immunology, highlighting the interdisciplinary nature of TILs research (**Figure 5F**).

**Table 4:** The top 10 journals contributed to publications on tumor-infiltrating lymphocytes.

Rank	Journal	Documents	Citations	H_index	M_index	IF/JCR (2023)	Country
1	*Cancer Immunology Immunotherapy*	103	3918	35	0.946	4.6/Q2	Germany
2	*Journal of Immunology*	73	7856	47	1.205	3.6/Q2	USA
3	*Cancers*	70	732	13	2.167	4.5/Q1	Switzerland
4	*Clinical Cancer Research*	57	7436	41	1.367	10/Q1	USA
5	*Journal of Immunotherapy*	56	2894	25	0.735	3.2/Q3	USA
6	*Oncoimmunology*	55	3000	30	2.308	6.5/Q1	United Kingdom
7	*Cancer Research*	48	3960	37	0.925	12.5/Q1	USA
8	*International Journal of Cancer*	48	2815	30	0.625	5.7/Q1	USA
9	*Anticancer Research*	46	1012	18	0.409	1.6/Q4	Greece
10	*Frontiers in Immunology*	44	970	14	1.273	5.7/Q1	Switzerland

IF = impact factor; JCR = Journal Citation Reports.

#### 3.2.5 Funding agencies

**Figure 6:**
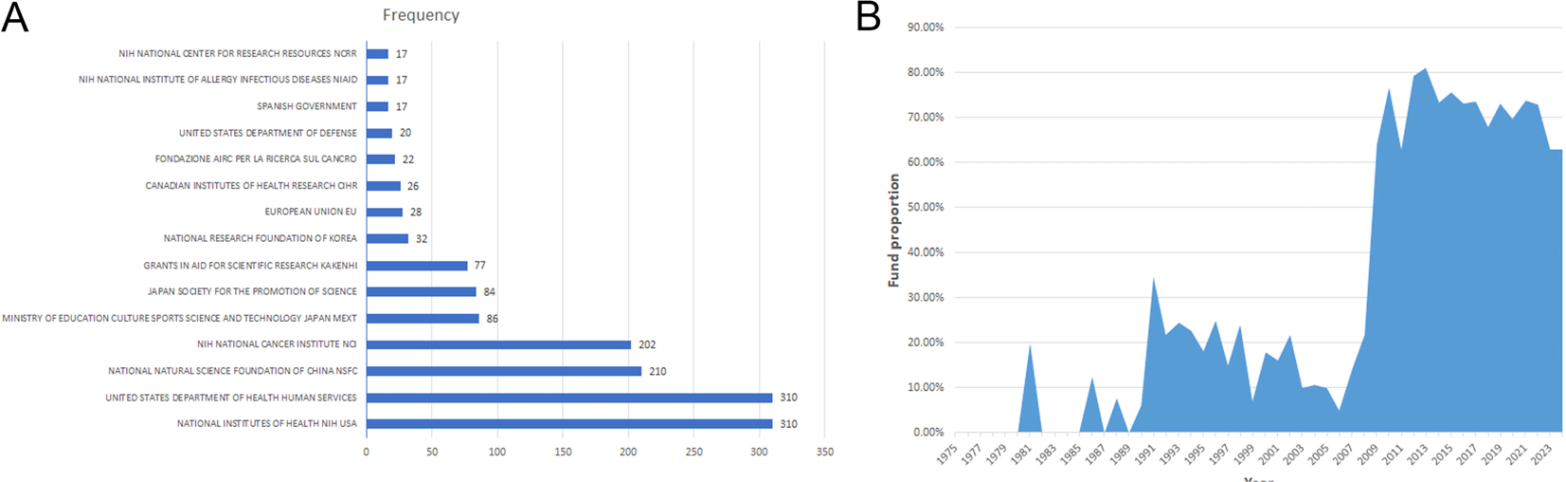
**(A)** The top 15 funding sources in TILs research. **(B)** The annual proportion of articles with funding in TILs research.

Funding support is evident, comprising 44.9% (1,159/2,584). The top two funders are the National Institutes of Health(310) and the United States Department of Health and Human Services (310) (**Figure 6A**). Since 2009, over 60% of publications have received financial support (**Figure 6B**), emphasizing funding’s pivotal role in driving research advancement. Moreover, articles from the USA received support at 61.18%, while those from China received a higher rate of 78.91%. Articles from Japan received support at 38.82%, from Italy at 49.07%, and from Germany at 51.45%, illustrating various national support in TILs research.

### 3.3 Analysis of keywords

**Figure 7:**
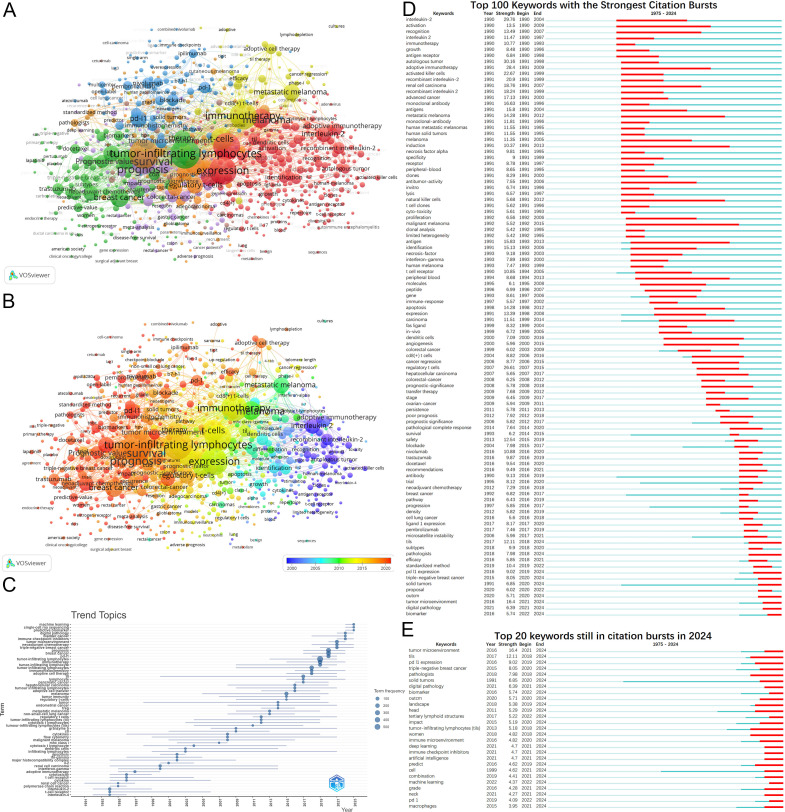
Analysis of keywords. **(A)** Keywords clustering. Keywords with frequencies exceeding 5 are clustered. Nodes represent keywords, with their size reflecting frequency. Lines denote connections between keywords. Colors indicate different keyword clusters. **(B)** Timeline view of keywords. The timeline elucidates not the inaugural emergence of keywords but rather their mean occurrence chronology. **(C)** Trend Topics from 1975 to 2024. **(D)** Top 100 keywords with the strongest citation bursts from 1975 to 2024. **(E)** Keywords still in citation bursts in 2024.

Keywords play a crucial role in encapsulating the themes of articles. Analysis of 5,851 keywords reveals a primary focus on diseases such as melanoma, breast cancer, colorectal cancer, lung cancer, and renal cell carcinoma. Keyword clustering identifies four major knowledge domains: adoptive cell therapy (red and yellow clusters); the prognostic value of TILs (green cluster); PD-1/PD-L1 immune checkpoint inhibitors and TILs (blue cluster); and prognostic studies of TILs across various cancers (orange and purple clusters) (**Figure 7A**). **Figure 7B** demonstrates current studies predominantly focus on the themes represented by the green, blue, orange, and purple clusters.

Analysis of annual high-frequency keywords (**Figure 7C**) also reveals a more nuanced shift in research focus. This shift has moved from early investigations into adoptive immunotherapy to exploring precision medicine, personalized treatments, and combination therapies. The rise of predictive biomarkers and machine learning highlights their growing importance as research hotspots.

Keyword burst detection similarly illustrates the evolution of research hotspots (**Figure 7D**). Burst analysis identifies 164 keywords with citation bursts(**Supplementary Material 5**), including interleukin-2 (29.76, 1990-2004), autologous tumor (30.16, 1991-1998), and adoptive immunotherapy (28.4, 1991-2009). Recent keywords with substantial bursts indicate future research trajectories, including PD-L1 expression, prediction, triple-negative breast cancer(TNBC), solid tumors, digital pathology, artificial intelligence, combination, biomarker, and tertiary lymphoid structures (**Figure 7E**).

### 3.4 Seminal literature - landmark research

Highly cited literature often signifies research that is both impactful and innovative within its field (**Supplementary Material 6**). Among the top 100 cited works, there are 14 reviews and 86 articles. The period between 2012 and 2016 experienced a notable surge in highly cited articles. **Table 5** details the top 20 articles, with studies by Rosenberg et al. from 1986 and 1988 highlighting the potential of TIL adoptive cell therapy in treating tumors in mice and metastatic melanoma patients, respectively (3, 32). These studies established a foundation for subsequent advancements in immunotherapy and achieved the highest citation counts (**Table 5**). Other highly cited articles primarily focus on critical technologies and methodologies related to TILs, including gene editing techniques, prognostic value, deep learning, and standardized assessment methods.

**Table 5:** The top 20 publications in number of total citations.

Title	Year	Citation	Author	Institution	Country	Source
Use of tumor-infiltrating lymphocytes and interleukin-2 in the immunotherapy of patients with metastatic melanoma - a preliminary-report	1988	2049	Rosenberg SA	National Institutes of Health (NIH)	USA	*New England Journal of Medicine*
The evaluation of tumor-infiltrating lymphocytes (tils) in breast cancer: recommendations by an international tils working group 2014	2015	2017	Salgado R	Institut Jules Bordet	Belgium	*Annals of Oncology*
Intraepithelial cd8 tumor-infiltrating lymphocytes and a high cd8/regulatory t cell ratio are associated with favorable prognosis in ovarian cancer	2005	1895	Sato E	Roswell Park Comprehensive Cancer Center	USA	*Proceedings of The National Academy of Sciences of The United States of America*
A new approach to the adoptive immunotherapy of cancer with tumor-infiltrating lymphocytes	1986	1636	Rosenberg SA	NIH National Cancer Institute (NCI)	USA	*Science*
Tumour-infiltrating lymphocytes and prognosis in different subtypes of breast cancer: a pooled analysis of 3771 patients treated with neoadjuvant therapy	2018	1286	Denkert C	Berlin Institute of Health	Germany	*Lancet Oncology*
Prognostic and predictive value of tumor-infiltrating lymphocytes in a phase iii randomized adjuvant breast cancer trial in node-positive breast cancer comparing the addition of docetaxel to doxorubicin with doxorubicin-based chemotherapy: big 02-98	2013	1235	Loi S	Institut Jules Bordet	Belgium	*Journal of Clinical Oncology*
Programmed cell death 1 ligand 1 and tumor-infiltrating cd8 t lymphocytes are prognostic factors of human ovarian cancer	2007	1196	Hamanishi J	Kyoto University	Japan	*Proceedings of The National Academy of Sciences of The United States of America*
Tumor-infiltrating cd8 lymphocytes predict clinical outcome in breast cancer	2011	1092	Mahmoud SMA	University of Nottingham	UK	*Journal of Clinical Oncology*
Gene-transfer into humans - immunotherapy of patients with advanced melanoma, using tumor-infiltrating lymphocytes modified by retroviral gene transduction	1990	1076	Rosenberg SA	Pathology Laboratory; Fred Hutchinson Cancer Research Center	USA	*New England Journal of Medicine*
Tumor infiltrating lymphocytes are prognostic in triple negative breast cancer and predictive for trastuzumab benefit in early breast cancer: results from the finher trial	2014	965	Loi S	Institut Jules Bordet	Belgium	*Annals of Oncology*
The prognostic influence of tumour-infiltrating lymphocytes in cancer: a systematic review with meta-analysis	2011	963	Gooden MJM	University of Groningen	Netherlands	*British Journal of Cancer*
Prognostic value of tumor-infiltrating lymphocytes in triple-negative breast cancers from two phase iii randomized adjuvant breast cancer trials: ecog 2197 and ecog 1199	2014	954	Adams S	New York University	USA	*Journal of Clinical Oncology*
Prognostic value of tumor infiltrating lymphocytes in the vertical growth phase of primary cutaneous melanoma	1996	871	Clemente CG	Albany Medical College	USA	*Cancer*
Identification of a human-melanoma antigen recognized by tumor-infiltrating lymphocytes associated with *in-vivo* tumor rejection	1994	850	Kawakami Y	National Institutes of Health (NIH)	USA	*Proceedings of The National Academy of Sciences of The United States of America*
Treatment of patients with metastatic melanoma with autologous tumor-infiltrating lymphocytes and interleukin-2	1994	806	Rosenberg SA	NIH National Cancer Institute (NCI)	USA	*Journal of The National Cancer Institute*
Tumor-infiltrating lymphocytes and response to neoadjuvant chemotherapy with or without carboplatin in human epidermal growth factor receptor 2-positive and triple-negative primary breast cancers	2015	777	Denkert C	Berlin Institute of Health	Germany	*Journal of Clinical Oncology*
Tumor-infiltrating lymphocyte grade is an independent predictor of sentinel lymph node status and survival in patients with cutaneous melanoma	2012	615	Azimi F	Melanoma Institute Australia	Australia	*Journal of Clinical Oncology*
B7-h1 expression on non-small cell lung cancer cells and its relationship with tumor-infiltrating lymphocytes and their pd-1 expression	2004	596	Konishi J	Hokkaido University	Japan	*Clinical Cancer Research*
Spatial organization and molecular correlation of tumor-infiltrating lymphocytes using deep learning on pathology images	2018	559	Saltz J	State University of New York (SUNY) System	USA	*Cell Reports*
Tumor-specific cytolysis by lymphocytes infiltrating human melanomas	1989	538	Topalian SL	National Institutes of Health (NIH)	USA	*Journal of Immunology*

Furthermore, the USA is the leading contributor with 44 articles, while other countries each contribute fewer than 8 articles. The top three institutions by publication volume are Harvard University (32), Institut Jules Bordet (25), and the National Institutes of Health (NIH) (25). The leading authors are Rosenberg SA (21), Loi S (11), and Denkert C (10). *Clinical Cancer Research* (12) and *the Journal of Clinical Oncology* (12) are the journals with the highest publication volumes.

### 3.5 Analysis of co-cited references

#### 3.5.1 Clusters and timeline of research

**Figure 8:**
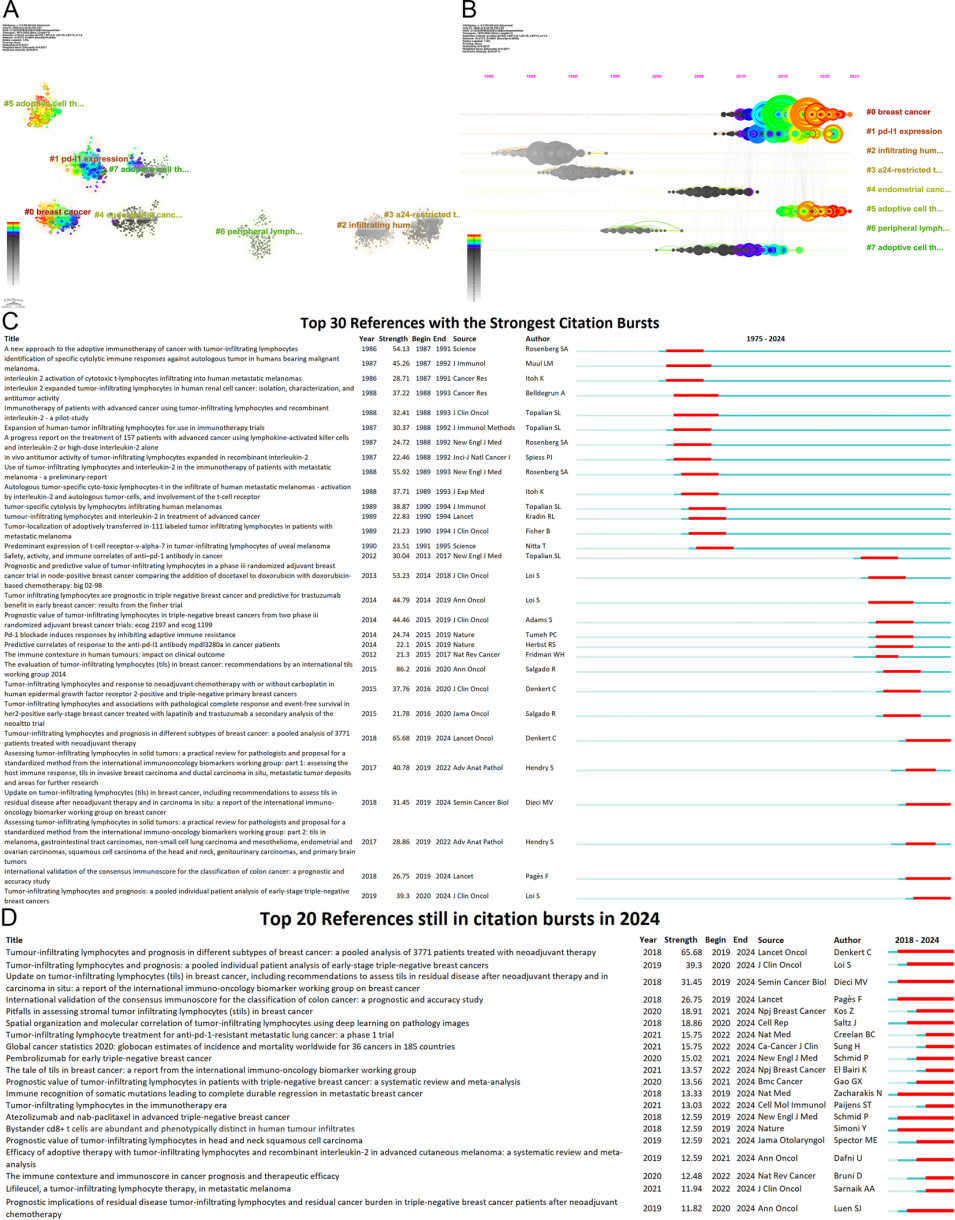
Analysis of references **(A)** Co-cited literature cluster in the TILs domain from 1975 to 2024. The average appearance time of the cluster is indicated in the lower-left corner. The transition from cooler to warmer color gradients visually depicts knowledge flow among these clusters. **(B)** Timeline View of co-cited literature Cluster. Each horizontal line is labeled with a cluster tag on its far right end. The size of nodes is proportional to the number of references, and the date of paper publication is indicated at the top of the figure. **(C)** Top 30 references with the strongest co-citation bursts from 1975 to 2024. The blue line represents the timeline spanning from 2008 to 2023, while the red line marks the specific time when each citation burst occurred. **(D)** Top 20 References still in citation bursts in 2024.

Co-citation reflects the referencing of two or more papers by other scholarly works, showcasing their interconnectedness within the research domain (33). Through co-citation analysis, it elucidates foundational knowledge and frontiers of academic research, enhancing our understanding of the field’s structure and evolution. CiteSpace was employed for co-citation analysis, resulting in 8 principal clusters(size>100) (**Figure 8A**) (**Supplementary Material 7**). The clustering results (Q = 0.8215, S = 0.9271) highlight reliability. The largest cluster is #0 breast cancer. Currently, breast cancer (#0), PD-L1 expression (#1), and adoptive cell therapy (#5) have progressively gained prominence (**Figure 8B**). **Table 6** details key references from these clusters.

**Table 6:** The top 10 co-cited references in #0, #1, #5 and #7 clusters.

Cluster	TI	Freq	Burst	Author	Year	Source
#0	The evaluation of tumor-infiltrating lymphocytes (tils) in breast cancer: recommendations by an international tils working group 2014	259	86.2(2016-2020)	Salgado R	2015	*Ann Oncol*
	Tumour-infiltrating lymphocytes and prognosis in different subtypes of breast cancer: a pooled analysis of 3771 patients treated with neoadjuvant therapy	188	65.68(2019-2024)	Denkert C	2018	*Lancet Oncol*
	Prognostic and predictive value of tumor-infiltrating lymphocytes in a phase iii randomized adjuvant breast cancer trial in node-positive breast cancer comparing the addition of docetaxel to doxorubicin with doxorubicin-based chemotherapy: big 02-98	123	53.23(2014-2018)	Loi S	2013	*J Clin Oncol*
	Tumor infiltrating lymphocytes are prognostic in triple negative breast cancer and predictive for trastuzumab benefit in early breast cancer: results from the finher trial	123	44.79(2014-2019)	Loi S	2014	*Ann Oncol*
	Prognostic value of tumor-infiltrating lymphocytes in triple-negative breast cancers from two phase iii randomized adjuvant breast cancer trials: ecog 2197 and ecog 1199	118	44.46(2015-2019)	Adams S	2014	*J Clin Oncol*
	Tumor-infiltrating lymphocytes and response to neoadjuvant chemotherapy with or without carboplatin in human epidermal growth factor receptor 2-positive and triple-negative primary breast cancers	117	37.76(2016-2020)	Denkert C	2015	*J Clin Oncol*
	Tumor-infiltrating lymphocytes and prognosis: a pooled individual patient analysis of early-stage triple-negative breast cancers	101	39.3(2020-2024)	Loi S	2019	*J Clin Oncol*
	Assessing tumor-infiltrating lymphocytes in solid tumors: a practical review for pathologists and proposal for a standardized method from the international immunooncology biomarkers working group: part 1: assessing the host immune response, tils in invasive breast carcinoma and ductal carcinoma *in situ*, metastatic tumor deposits and areas for further research	100	40.78(2019-2022)	Hendry S	2017	*Adv Anat Pathol*
	Update on tumor-infiltrating lymphocytes (tils) in breast cancer, including recommendations to assess tils in residual disease after neoadjuvant therapy and in carcinoma *in situ*: a report of the international immuno-oncology biomarker working group on breast cancer	92	31.45(2019-2024)	Dieci MV	2018	*Semin Cancer Biol*
	Tumor-infiltrating lymphocytes and associations with pathological complete response and event-free survival in her2-positive early-stage breast cancer treated with lapatinib and trastuzumab a secondary analysis of the neoaltto trial	68	21.78(2016-2020)	Salgado R	2015	*Jama Oncol*
#1	Cancer statistics, 2017	77	9.76(2019-2024)	Siegel RL	2021	*CA-Cancer J Clin*
	Assessing tumor-infiltrating lymphocytes in solid tumors: a practical review for pathologists and proposal for a standardized method from the international immuno-oncology biomarkers working group: part 2: tils in melanoma, gastrointestinal tract carcinomas, non-small cell lung carcinoma and mesothelioma, endometrial and ovarian carcinomas, squamous cell carcinoma of the head and neck, genitourinary carcinomas, and primary brain tumors	76	28.86(2019-2022)	Hendry S	2017	*Adv Anat Pathol*
	PD-1 blockade induces responses by inhibiting adaptive immune resistance	66	24.74(2015-2019)	Tumeh PC	2014	*Nature*
	Safety, Activity, and Immune Correlates of Anti–PD-1 Antibody in Cancer	62	30.04(2013-2017)	Topalian SL	2012	*New Engl J Med*
	Predictive correlates of response to the anti-PD-L1 antibody MPDL3280A in cancer patients	59	22.1(2015-2019)	Herbst RS	2014	*Nature*
	Classifying Cancers Based on T-cell Infiltration and PD-L1	51	20.83(2017-2020)	Teng MWL	2015	*Cancer Res*
	Nivolumab versus Docetaxel in Advanced Nonsquamous Non–Small-Cell Lung Cancer	48	16.82(2016-2020)	Borghaei H	2015	*New Engl J Med*
	Association of PD-1, PD-1 Ligands, and Other Features of the Tumor Immune Microenvironment with Response to Anti–PD-1 Therapy	47	17.59(2015-2019)	Taube JM	2014	*Clin Cancer Res*
	Towards the introduction of the ‘Immunoscore’ in the classification of malignant tumors	47	17.59(2015-2019)	Galon J	2014	*J Pathol*
	The immune contexture in human tumors: impact on clinical outcome	42	21.3(2015-2017)	Fridman WH	2012	*Nat Rev Cancer*
#5 and #7	International validation of the consensus Immunoscore for the classification of colon cancer: a prognostic and accuracy study	72	26.75(2019-2024)	Pagès F	2018	*Lancet*
	Immune recognition of somatic mutations leading to complete durable regression in metastatic breast cancer	36	13.33(2019-2024)	Zacharakis N	2018	*Nat Med*
	Durable Complete Responses in Heavily Pretreated Patients with Metastatic Melanoma Using T-Cell Transfer Immunotherapy	35	19.48(2012-2016)	Rosenberg SA	2011	*Clin Cancer Res*
	Bystander CD8+ T cells are abundant and phenotypically distinct in human tumour infiltrates	34	12.59(2019-2024)	Simoni Y	2018	*Nature*
	PD-1 identifies the patient-specific CD8+ tumor-reactive repertoire infiltrating human tumors	31	11.58(2015-2019)	Gros A	2014	*J Clin Invest*
	Understanding the tumor immune microenvironment (TIME) for effective therapy	31	11.49(2020-2024)	Binnewies M	2018	*Nat Med*
	Clinical Responses in a Phase II Study Using Adoptive Transfer of Short-term Cultured Tumor Infiltration Lymphocytes in Metastatic Melanoma Patients	30	17.66(2010-2015)	Besser MJ	2010	*Clin Cancer Res*
	Tumor-infiltrating lymphocyte treatment for anti-pd-1-resistant metastatic lung cancer: a phase 1 trial	29	15.75(2022-2024)	Creelan BC	2021	*Nat Med*
	Randomized, prospective evaluation comparing intensity of lymphodepletion before adoptive transfer of tumor-infiltrating lymphocytes for patients with metastatic melanoma	28	12.32(2018-2021)	Goff SL	2016	*J Clin Oncol*
	The immune contexture in cancer prognosis and treatment	28	11.94(2020-2022)	Fridman WH	2017	*Nat Rev Clin Oncol*

The central components of breast cancer (#0) primarily address the application of TILs in the treatment and prognosis of breast cancer, predominantly featured in high-impact journals such as *Annals of Oncology, Lancet Oncology*, and *Journal of Clinical Oncology*. Several studies have revealed the prognostic and predictive value of TILs across various breast cancer subtypes (12, 34–36), particularly TNBC (37–40), encompassing both chemotherapy-treated and untreated patients (38). Furthermore, numerous studies have investigated the role of TILs as a significant predictor of neoadjuvant chemotherapy response to breast cancer (9, 10). Elevated TIL levels in TNBC are generally associated with improved responses to neoadjuvant chemotherapy and enhanced survival benefits (41). Additionally, the presence of TILs in residual disease following neoadjuvant chemotherapy also correlates with a better prognosis (9).

The principal themes of clusters #5 and #7 revolve around adoptive cell therapy, with cluster #7 being established earlier, having an average emergence year of 2009, while cluster #5 emerged around 2018. Analyzing the clusters reveals a trajectory of TIL therapy evolving from initial exploration to personalized treatment approaches. Seminal studies conducted by Rosenberg SA and his team at the National Institutes of Health in 1986 and 1988 illustrated the antitumor potential of TILs combined with IL-2 (3, 32). Subsequent advancements—such as the optimization of TIL isolation and expansion, chemotherapeutic preconditioning (e.g., lymphocyte-depleting chemotherapy), and advancements in gene engineering—have markedly improved the clinical effectiveness of TIL therapy (42, 43). Over the past three decades, numerous clinical centers have validated the feasibility of this therapy (44–47). As research has advanced, the notion of personalized treatment has become increasingly prominent. Research by Goff SL has suggested the need for personalized lymphodepletion regimens tailored to individual patient profiles (47).

Articles on PD-L1 expression (#1) reveal pivotal insights into the relationship between TILs and PD-L1, predominantly featuring in prestigious journals such as the *New England Journal of Medicine*. Over the past decade, the introduction of immune checkpoint inhibitors, particularly PD-1/PD-L1 inhibitors, has significantly reshaped the landscape of cancer immunotherapy. Since 2015, there has been an explosion of research on the expression of PD-1 and PD-L1 in tumors and their interactions with TILs. Research indicates that tumors with high TILs frequently also show elevated PD-L1 expression (48–50). Extensive studies have established that elevated levels of both TILs and PD-L1 expression correlate with a favorable prognosis in cancers(e.g., breast cancer), and with the efficacy of neoadjuvant chemotherapy (11, 49, 50). Additionally, PD-1 expression helps identify tumor-reactive TILs (51), and the presence of TILs is closely linked to the effectiveness of immune checkpoint inhibitor therapies. These developments have accelerated research on TILs as biomarkers for predicting immunotherapy outcomes (52–54) and combined treatment approaches (21). In particular, some “cold” tumors, such as non-small-cell lung cancer, can be transformed into “hot” tumors through combination therapies, enhancing their sensitivity to immune checkpoint inhibitors (21). Moreover, by blocking the PD-L1/PD-1 pathway, TIL suppression can be mitigated, leading to increased TIL numbers in the TME and a restoration of their tumor-fighting capabilities (22).

#### 3.5.2 Burst analysis

The burst of citations has provided a valuable means for observing the evolution of research focal points (**Figure 8C**). A total of 499 references exhibited citation bursts (**Supplementary Material 7**). As of 2024, 82 references are still experiencing citation bursts (**Figure 8D**), with 35 in cluster #0 (breast cancer), 34 in cluster #5 (adoptive cell therapy), and 4 in cluster #1 (PD-L1 expression). The primary topics include the prognostic significance of TILs in melanoma, breast cancer, colorectal cancer, and head and neck squamous cell carcinoma; the role of TILs in predicting the efficacy of neoadjuvant chemotherapy; the relationship between TILs and PD-L1; TIL assessment; TIL-related biomarkers; TILs in the TME; and tertiary lymphoid structures. These topics represent key current and emerging focuses in TILs research.

## 4 Discussion

### 4.1 General information

Over the past two decades, research on TILs has evolved through three distinct phases, with a notable acceleration since 2015. This growth is closely linked to the emergence of PD-1/PD-L1 immune checkpoint inhibitors, which, as groundbreaking tumor treatments, have significantly boosted TILs-related research. Researchers from 78 countries and regions have contributed to TIL studies, but only a few nations dominate the field. The USA leads in both the volume of publications and impact metrics, establishing itself as a dominant player in the field. This leadership is attributed not only to its strong research infrastructure and technological innovations but also to a long-standing tradition of interdisciplinary collaboration and abundant resource support. However, international collaboration needs to be enhanced, which could help countries with lower research impact, such as South Korea, to elevate their influence. By fostering global partnerships, these nations could tap into cross-border research networks, integrating resources and talent to accelerate the development and application of novel therapies. China’s rapid advancement in TIL research is another noteworthy trend. Supported by strong national policies and an innovation-driven development strategy, China has made significant strides over the past decade, particularly in research on the therapeutic and prognostic value of TILs and immune checkpoint inhibitors. This progress is evident in its prolific output, signaling that Chinese research institutions are gradually transitioning from early theoretical work to clinical applications, with a focus on patient-centered immunotherapy. However, compared to other nations, China still faces challenges in foundational scientific innovation and international collaboration.

The NCI excels in both publications and citations, particularly for its pivotal contributions to adoptive cell therapy in melanoma. Institutions such as the Jules Bordet Institute, the German Cancer Consortium, the National Institutes of Health, and Heidelberg University are renowned for their high-quality research. Conversely, institutions like Sun Yat-sen University, the University of Ulsan, and Fudan University need to enhance the quality of their publications. Institutions actively contributing to the field at present include the University of Helsinki, the University of Texas System, Harvard University, and Sun Yat-sen University. Targeting these active institutions could facilitate the establishment of efficient collaborative networks. Affiliated with the NCI, Steven A. Rosenberg is unquestionably a seminal figure in the field, particularly renowned for his work in adoptive cell therapy and melanoma. Researchers such as Lee HJ, Hwu P, and Gong G are encouraged to enhance the impact of their publications. Noteworthy recent contributors include Lee HJ, Loi S, Roberto Salgado, Giuseppe Floris, Yoshinao Oda, and Marco Donia.

Academic dissemination in the TILs research domain is highly concentrated, with high-impact journals playing a dominant role. *Cancer Immunology Immunotherapy* has published the largest number of articles, consistently addressing topics such as PD-L1, prognosis, and breast cancer. *The Journal of Immunology*, with its rich historical legacy, has made indispensable contributions to the early stages of TILs research. Additionally, focusing on contemporary themes within high-impact journals facilitates the exploration of novel applications for TILs. Recent prominent contributors include *Frontiers in Immunology*, *Cancers*, *Frontiers in Oncology*, *Journal for Immunotherapy of Cancer*, and *Scientific Reports*. These journals predominantly feature articles on PD-L1, prognosis, breast cancer, neoadjuvant chemotherapy, biomarkers, and adjuvant chemotherapy. Submitting relevant articles to these publications is a commendable choice. Overlay maps illustrate the interdisciplinary nature of TILs research.

The critical role of funding in TILs research has become increasingly evident, particularly since the surge in research funding after 2009, which has significantly accelerated scientific progress in this field. In the United States, primary funding bodies such as the National Institutes of Health (NIH) and the Department of Health and Human Services (HHS) have been driving the nation’s ongoing contributions to TILs research. In contrast, China has seen a rapid rise in its financial investment in TILs research, closely aligned with its strategic emphasis on promoting technological innovation and foundational research, particularly in cutting-edge areas like cancer immunotherapy. However, the disparities in funding across nations highlight the global unevenness of this research area. Future studies should focus on enhancing international collaboration and financial integration to expedite the development and application of TILs technologies.

### 4.2 Hotspots and Frontiers of TILs

The analysis of keywords and references indicates that “adoptive cell therapy,” “the prognostic value of TILs,” and “immune checkpoint inhibitors and TILs” are central themes in current and prospective research. Key research hotspots include the ongoing investigation of TILs in breast cancer, colorectal cancer, head and neck carcinoma, and other solid tumors; combination therapies; tumor neoantigens; gene editing; dominant population selection of TILs therapy; TILs in TME; TILs-related biomarkers; TILs in predicting the efficacy of neoadjuvant chemotherapy and immunotherapy; PD-1/PD-L1; TIL-based patient stratification; tertiary lymphoid structures; and TILs evaluation(digital pathology and artificial intelligence).

#### 4.2.1 TILs therapy

##### 4.2.1.1 Optimization of TILs therapy

The application of TILs therapy in melanoma treatment has made significant advancements, with numerous clinical trials demonstrating a high response rate in patients with advanced melanoma (44, 55, 56). Research is currently extending to other cancer types, including breast, lung, cervical, and colorectal cancers (21, 47, 57–60). However, its clinical application faces several challenges, most notably the highly immunosuppressive TME. Combining TIL therapy with immune checkpoint inhibitors like PD-L1/PD-1 to counteract the immunosuppressive TME represents a significant research direction. Studies have demonstrated that such combinations can improve therapeutic efficacy and increase overall patient survival (21, 61). Furthermore, some clinical studies indicate that TIL therapy may provide a new treatment option for patients resistant to PD-L1 inhibitors (21, 55).

Genetic modification of TILs is also a critical approach. For instance, Michael R. Schlabach’s team utilized CRISPR/Cas9 technology to knock out specific genes (SOCS1 gene) in TILs to enhance their functionality (62). Moreover, advancements in high-throughput sequencing and epitope prediction algorithms have brought an emerging research direction that involves developing personalized TIL therapies based on neoantigen information (58, 63–65), with studies indicating that neoantigen-specific T cells can provoke potent antitumor responses in certain patients (58). For instance, the team led by Parkhurst successfully combined T cells targeting specific neoantigens with checkpoint blockade therapy, resulting in complete and durable tumor regression (55, 63).

Moreover, the intricate production process and high costs associated with TIL therapy present significant barriers to widespread adoption. Consequently, reducing costs and improving accessibility are critical challenges. The integration of novel materials offers promising avenues (66, 67), such as hydrogels with high drug-loading capacity, sustained release, and excellent biocompatibility (68).

##### 4.2.1.2 Dominant population selection

The efficacy of TIL therapy varies significantly among individuals, making the prediction and optimization of treatment outcomes a critical issue and hotspot. Addressing these challenges requires a thorough understanding of the mechanisms underlying adoptive cell therapy. Research on biomarkers related to the identification of tumor-reactive TILs, such as CD39 (69) and PD-1 (51), is ongoing. Additionally, exploring the heterogeneity and functional characteristics of TILs across different tumors and their subpopulations, through technologies like single-cell RNA sequencing, spatial transcriptomics, and mass spectrometry (69), can elucidate various immune escape mechanisms (13). For example, David König’s team collected T cells from multiple tumor sites before treatment to analyze tumor heterogeneity, aiming to enhance antitumor activity and overcome resistance to TIL therapy (14). Moreover, an in-depth examination of patient-specific tumor characteristics and immune status, including TIL types, functions, and dynamic changes, is crucial for optimizing patient selection and personalizing TIL treatment strategies. For instance, Barras and David’s team discovered that the clinical response of melanoma to TIL-ACT is associated with a pre-existing, identifiable network of CD8+ TIL and myeloid cells (7).

Despite the notable success of TIL therapy in treating specific cancers like melanoma, its application to other malignancies remains fraught with challenges. Future crucial research issues include immune escape mechanisms, TIL functional decline, and the efficiency of *in vivo* expansion. A deeper understanding of the complexity of the TME and the dynamic roles of TILs within it, alongside the integration of various immunomodulatory strategies, may pave the way for personalized and holistic immunotherapy.

#### 4.2.2 Prognostic value of TILs

The prognostic value of TILs is unequivocal, particularly in TNBC (37–40). TILs also serve as a predictive marker for positive chemotherapy response in these patient populations (9, 10). Furthermore, the prognostic role of TILs in other solid tumors, such as head and neck squamous cell carcinoma, oropharyngeal cancer, and laryngeal cancer, is becoming increasingly well-understood and will become a research hotspot in the future (70, 71). For instance, the Society for Immunotherapy of Cancer utilizes the consensus Immunoscore assay to evaluate tumor-infiltrating T-cell counts to predict recurrence risk in patients with stage I-III colon cancer (72).

With the emergence of immune checkpoint inhibitors such as PD-1/PD-L1 inhibitors, integrating PD-L1 expression and TIL characteristics for disease prognosis has gained considerable attention currently (11, 73). Several studies have delved into combining these markers with relevant gene expression levels to predict overall survival in cancer patients (74, 75). Moreover, numerous studies have shown that the presence and activity of TILs are closely linked to the efficacy of PD-1/PD-L1 checkpoint inhibitors. High-density TILs may indicate a more favorable response to ICI therapy (19).

Additionally, aside from PD-L1, LAG3+ (a negative immune regulator) is also highly expressed in TILs (76, 77), with ongoing research examining its potential as a biomarker for predicting immune therapy responses and prognosis (26). Furthermore, serving as local immune centers, numerous studies have highlighted tertiary lymphoid structures as a favorable prognostic factor for cancer (78), particularly breast cancer (8, 79). The interplay between tertiary lymphoid structures, TILs, and clinical outcomes is being thoroughly investigated across different tumor types (80, 81).

#### 4.2.3 TIL-based patient stratification

Consequently, as key biomarkers for prognosis and therapeutic response in certain cancers, TIL-based patient stratification is currently a significant area of research, aiming to reduce overtreatment, minimize potential toxicity, and address cost considerations (82). For example, identifying TNBC patients who might benefit from less aggressive chemotherapy is essential (83, 84). Some studies have proposed TIL cutoff values to stratify chemotherapy strategies for stage I TNBC patients (85); however, controversy remains regarding the definition of these cutoff values.

Moreover, using TILs, PD-L1, and other biomarkers to identify patients likely to benefit from ICI therapy is being hotly investigated (23–25, 82). For instance, Eftychia Chatziioannou’s team discovered that in untreated metastatic melanoma, an electronic TIL score ≤12.2% could predict poor survival outcomes in patients undergoing anti-PD-1 therapy (86). Similarly, Carmine Valenza’s team identified that PD-L1 CPS positivity and/or TIL scores ≥1% could forecast skin metastases in 57% of patients with chest wall breast cancer (87).

In summary, the prognostic value of TILs in specific cancers is well established, and the concept of utilizing TILs for patient stratification is increasingly acknowledged. Developing models that integrate TILs and other biomarkers to predict treatment response and long-term prognosis remains a.

#### 4.2.4 Evaluation of TILs

The significant role of TILs in prognosis and therapy has led to increased focus on TIL assessment. Recent advancements in digital pathology and artificial intelligence have facilitated the automation of TIL assessment, improving its efficiency, objectivity, and consistency (15, 16). These technological innovations also facilitate the detailed evaluation of the functional characteristics and spatial distribution of TILs (15, 17–19), such as the AI-driven TIL spatial analysis developed by Sehhoon Park’s team (18), positioning it as a current and future research hotspot. For example, Joel Saltz’s team employed convolutional neural networks to analyze H&E-stained digital slides (17). Additionally, the integration of multidimensional data (e.g., genomics, transcriptomics, proteomics) creates a panorama of TILs within the TME. This approach elucidates their complex interaction networks and supports the identification of novel biomarkers and therapeutic targets (20), marking a significant area of future research.

## 5 Strengths and limitations

Several limitations must be acknowledged: 1) Solely incorporating WOSCC data could potentially introduce selection bias. 2) Bibliometric methodologies, reliant on natural language processing, may entail errors. Nevertheless, this research remains valuable for readers interested in understanding the current status, focal points, and trends in the field of TILs.

## 6 Conclusion

This study employs bibliometric analysis to map the evolution of knowledge over the past 49 years, addressing the four key questions posed in the Introduction:

### 6.1 What is the overarching trajectory of research concerning TILs?

TIL research has evolved through three phases, with rapid growth since 2015. This trajectory reflects a transition from foundational studies to translational and clinical applications, particularly in cancer immunotherapy, highlighting the increasing integration of TILs into therapeutic and prognostic strategies.

### 6.2 Which nations, institutions, journals, authors, and funders exert the most significant influence within this research purview?

The USA leads in publication volume and scientific impact, with institutions such as the NCI and Harvard University playing pivotal roles. Steven A. Rosenberg stands out as a seminal contributor, particularly in adoptive cell therapy. Influential journals like Cancer Immunology Immunotherapy and Journal for Immunotherapy of Cancer serve as key platforms for disseminating high-impact findings. Funding agencies such as the NIH have been instrumental in supporting breakthroughs in this field.

### 6.3 In which primary disease areas are TILs most commonly applied?

TIL research has focused predominantly on melanoma, breast cancer, and colorectal cancer. These disease areas underscore the prognostic and therapeutic relevance of TILs, especially in immune checkpoint inhibitors and neoadjuvant chemotherapy.

### 6.4 What are the pivotal research themes within this domain? Which studies are regarded as seminal milestones? What are the current research hotspots? What are the potential future research directions?

Key themes include adoptive cell therapy, the prognostic value of TILs, PD-1/PD-L1 immune checkpoint inhibitors and TILs, and prognostic studies of TILs across various cancers. Foundational studies by Rosenberg et al. established the therapeutic potential of TILs, setting the stage for advancements in adoptive cell therapy. Current hotspots focus on “adoptive cell therapy,” “the prognostic value of TILs,” and “immune checkpoint inhibitors and TILs”. Future research should synthesize insights and methodologies from immunology, molecular biology, computational biology, and engineering to thoroughly investigate the mechanisms of TILs in tumors, establish standardized assessment methods, and discover novel biomarkers and clinical prediction models. Such efforts could potentially yield new breakthroughs and hope for cancer treatment.

By systematically addressing these questions, this study not only elucidates the development and current status of TIL research but also identifies key opportunities for advancing this promising field. Additionally, using CiteSpace, VOSviewer, and R-bibliometrix underscores the potential for reproducibility and validation with new data, based on selected computational attributes.

## Data Availability

The original contributions presented in the study are included in the article/**Supplementary Material**. Further inquiries can be directed to the corresponding authors.
