# Supplementary material for: The global trends and distribution in tumor-infiltrating lymphocytes over the past 49 years: bibliometric and visualized analysis
Source: Front Immunol. 2025 Jan 6;15:1511866. doi: 10.3389/fimmu.2024.1511866 (PMC11743541; doi:10.3389/fimmu.2024.1511866)
Supplement: Supplementary file 1 [file Table1.docx]

**The Global Trends and Distribution in Tumor-infiltrating Lymphocytes over the past 49 Years：Bibliometric and visualized analysis**

**Beibei Wu**

**The detailed information of “3.1 Materials and Methods”**

**1 Data sources and collection**

This study utilized the Web of Science(WOS) as its primary database due to its coverage of over 12,000 academic journals, housing more than 74.8 million scholarly records across 254 disciplinary fields, with 1.5 billion references since 1900^33^. It encompasses a significant corpus of high-quality academic literature, providing robust data support, and is widely used by researchers. Additionally, compared to databases such as Scopus, Medline, and PubMed, WOS is well-suited for various bibliometric analysis tools^34^ (for example, citespace) and serves as a principal data source for bibliometric analysis.

The search strategy used was: TI=(Lymphocytes, Tumor-Infiltrating OR Tumor-Infiltrating Lymphocyte OR Lymphocyte, Tumor-Infiltrating OR Tumor-Infiltrating Lymphocytes OR Tumor Infiltrating Lymphocyte OR Infiltrating Lymphocytes, Tumor OR Infiltrating Lymphocyte, Tumor OR Lymphocytes, Tumor Infiltrating OR Lymphocyte, Tumor Infiltrating OR Tumor Infiltrating Lymphocytes).

**2The overview of Bibliometric Analysis and Visualization**

| **Tool** | **Function** | **Default Parameter Configuration** | **Adjustment of other Parameters** | |
| --- | --- | --- | --- | --- |
| Microsoft Excel 2019 | line graphs (Figure 1A);  bar graphs (Figure 6);  all tables | | | |
| Citespace(https://citespace.podia.com/download, R6.1.6) | overlayMaps of journals  (Figure 5D) | Enable the overlay function for journals in the “JCR Journal Maps” under the “OverlayMaps” menu, and merge connections using the “Z-score” functionality. | | |
|  | Institutions:  Institutional collaboration network (Figure 3B);  burst analysis(Figure 3C) | Timespan=1975–2024;  Years per slice=1;  Links:Strength(Cosine), Scope(Within Slices);  Selection criteria:g-index (k=25), Pruning :no;  Show cluster labels:log-likelihood ratio(LLR) | 1. Node type = institution; | |
|  | Authors:  Collaboration network of authors (Figure 4C);  burst analysis(Figure 4B) |  | 1. Node type = author; | |
|  | Keywords:  burst analysis(Figure 7D, Figure 7E); |  | 1. Node type = keywords; | |
|  | co-cited references:  cluster analysis(Figure 8A, Figure 8B),  burst analysis(Figure 8C, Figure 8D) |  | 1. Node type = reference;  2. Due to the absence of keywords in Web of Science (WOS) prior to 1990, terms were extracted from titles, and each co-citation cluster was named using title words with the highest log-likelihood ratio.  3.Display the largest K clusters:8 | |
| VOSviewer  (https://www.vosviewer.com/, R1.6.18) | Journal:  Co-occurrence of journals(Figure 5A)  Map of co-cited journals (Figure 5E) | Counting method: Full counting | No | |
|  | Co-occurrence of keywords  (Figure 7A);  Timeline view of keywords(Figure 7B) |  | No | |
|  | Co-occurrence of journal-keyword  (Figure 5D) |  | Choose threshold(Minimum number of occurrences of a keyword/journal:10)  Max. lines:500 | |
|  | Keywords contributions across different countries and institutions in TILs research. (Figure 2C, Figure 2D, Figure 2E, Figure 2F,Figure 3A) |  | No(refer to "3.1 Network Overlay Analysis”) | |
| Scimago Graphica  (Setup 1.0.35) | Geographic visualization of co-authorship network:  countries  (Figure 2A) | Firstly, export the GML format file of co-authorship network from VOSviewer;  Secondly, configure the parameters:  Size: document;  Color: citations; | Label:country, document, citations;  Marks:disks(layout:map); | |
| R-bibliometrix and R-studio  (version R 4.3.0) | H_index and M_index:journal, author  (Table 3, Table 4);  Number of documents from corresponding authors' countries(Figure 2B);  Core Sources by Bradford's Law(Figure 5B);  Trend Topics (Figure 7C) | Importing data into R-bibliometrix yields results directly. | | |
|  | Yearly occurrences:top 30 institutions, authors, journals (Figure 3D; Figure 4A; Figure 5C); | 1. Import the plain text data exported from WOS into R-Bibliometrix, and export it as a CSV file named “Bibliometrix-Export-File-2024-05-10”.  2. Use R-studio to read the “Bibliometrix-Export-File-2024-05-10.csv” and write the corresponding code to compute the results. | | code |

**3.**

Moreover, the implementation of two functionality is intricate, requiring a flexible understanding of the structure of the WoS dataset and the principles of VOSviewer.

**3.1 Network Overlay Analysis**

VOSviewer enables the superimposition of specific data subsets onto the overall dataset, such as constructing a network of keyword co-occurrences for a given country across the entire network. In this study, figure 2C, figure 2D, figure 2E, figure 2F, and figure 3A utilized this functionality. Taking Figure 2C as an illustrative example:

Firstly, during the initial data collection phase, besides gathering all literature comprehensively, literature datasets categorized by the top countries and institutions were also collected.

Secondly, employing VOSviewer to construct the overall keyword co-occurrence network of tumor-infiltrating lymphocytes research, followed by clicking "save" to preserve the map file and network file from VOSviewer, saved as map1.csv and net1.txt.

Thirdly, utilizing VOSviewer to import data from USA and generate the keyword co-occurrence network for USA. Subsequently, clicking "save" saves the two files map2.csv and net2.txt.

Fourthly, opening map1.csv in Excel, where, due to this study's utilization of color variation to denote proportional sizes, the 'score' field in the map file represents color. Thus, creating a new column "score<keyword Occurrences>" and utilizing Excel's "VLOOKUP" function to match the keyword frequencies from map2.csv to the "score<keyword Occurrences>" column in map1.csv.

Fifthly, creating a new column "score<keyword scale>" in map1.csv, dividing the frequencies in "score<keyword Occurrences>" by the total keyword occurrences in the 'weight<Occurrences>' column, obtaining proportions, and filling in the "score<keyword scale>" column. Ultimately, this yields the contribution proportions of tumor-infiltrating lymphocytes research keywords from USA relative to the overall tumor-infiltrating lymphocytes research.

Sixthly, saving the modified map1.csv as a "Text (tab delimited)(*.txt)" file, named map1.txt.

Seventhly, using VOSviewer's "open" function to import map1.txt and net1.txt, switching to the "Overlay Visualization" interface, selecting the 'scores' function in the Visualization Operations interface, and choosing the newly added "score<keyword scale>" for colors, selecting "Rainbow" for the colors option, thereby obtaining a visual representation of the contribution proportions of tumor-infiltrating lymphocytes research keywords from USA relative to the overall tumor-infiltrating lymphocytes research.

Other overlays based on VOSviewer operate similarly.

**2.2 Co-occurrence of journal-keyword**

The co-occurrence network of journal-keyword is dependent on plain text data exported from WoS. This data comprises 68 fields, including DA(export time of document), SO (source), DE (author keywords), and ID (keywords Plus), which are crucial for constructing the co-occurrence network of journal-keyword.

Firstly, delete all “DA fields”.

Secondly, replace "ID" with "DA".

Thirdly, replace "SO" with "ID".

Fourthly, utilize VOSviewer's “co-occurrence” function.

Note: It should be clarified that the keywords in this co-occurrence network of journal-keyword consist solely of author keywords, distinct from the content of keywords in Co-occurrence of keywords(Figure 7A). VOSviewer's “co-occurrence” function is unique in its ability to identify co-occurrences between two fields,.

**2.3** Code of Yearly occurrences figure

## Yearly occurrences of the top 30 institutions

setwd("D:\\桌面\\肿瘤浸润淋巴细胞\\R分析结果")

library(reshape2)

library(tidyverse)

library(bibliometrix)

#数据读入法

Bw<-read.csv("Bibliometrix-Export-File-2024-05-10.CSV")#biblioshiny获取

synonyms <- c("HARVARD UNIVERSITY; HARVARD MEDICAL SCHOOL","UNIVERSITY OF TEXAS SYSTEM; UTMD ANDERSON CANCER CENTER","UNIVERSITY OF MICHIGAN; UNIVERSITY OF MICHIGAN SYSTEM")

topKW=KeywordGrowth(Bw, Tag = "AU_UN", sep = ";", top=30, cdf=FALSE,synonyms=synonyms)

topKW2=melt(topKW, id='Year')

#折线图

ggplot(topKW2,aes(Year,value, group=variable, color=variable))+geom_line()+theme_bw()

#标准化

library(plyr)

library(scales)

data <- ddply(topKW2, .(variable),transform,rescale = rescale(value))

#rescale()函数内默认参数to = c(0,1)，所以标准化范围也可以自定义

#热图

library("viridis")#渐变色

ggplot(data, aes(Year, variable)) +

geom_tile(aes(fill =rescale),colour = "white") +

#scale_fill_distiller(palette="Spectral",name="value") +

#scale_fill_gradientn(values = seq(0,1,0.2),colours = c('cyan','blue','green','orange','red'),name = "value")+

#scale_fill_viridis(option = "A",name = "value")+

scale_fill_viridis(option = "B",name = "value")+

#scale_fill_viridis_c(name = "value")+

coord_fixed(ratio=1)+

scale_x_continuous(expand = c(0,0))+#这个可以去掉与X轴间隙

scale_x_continuous(expand=c(0,0),

breaks=seq(1975, 2023, by=1)) +

theme_bw()+

theme(axis.text.x = element_text(angle=90,vjust = 0.5, hjust = 0.5))+

theme(axis.text= element_text(size = 9,family="serif"))+

theme(axis.title=element_text(size=14,face="bold"))+

labs(x = "Year", y = "Institutions", title = "Institutions Distribution Over Time")+

theme(plot.title = element_text(size = 15,hjust = 0.5,face="bold"))+

theme(legend.key.width=unit(3,'mm'),legend.key.height=unit(3,'cm'))+

theme(legend.title = element_text(size = 10,family="serif"))+

theme(panel.grid.major =element_blank(),

panel.grid.minor = element_blank(),

panel.background = element_blank(),#去除背景

panel.border = element_blank())#去除边框

## Yearly occurrences of the top 30 authors

setwd("D:\\桌面\\肿瘤浸润淋巴细胞\\R分析结果")

library(reshape2)

library(tidyverse)

library(bibliometrix)

#数据读入法

Bw<-read.csv("Bibliometrix-Export-File-2024-05-10.CSV")#biblioshiny获取

topKW=KeywordGrowth(Bw, Tag = "AU", sep = ";", top=30, cdf=FALSE)

topKW2=melt(topKW, id='Year')

#折线图

ggplot(topKW2,aes(Year,value, group=variable, color=variable))+geom_line()+theme_bw()

#标准化

library(plyr)

library(scales)

data <- ddply(topKW2, .(variable),transform,rescale = rescale(value))

#rescale()函数内默认参数to = c(0,1)，所以标准化范围也可以自定义

#热图

library("viridis")#渐变色

ggplot(data, aes(Year, variable)) +

geom_tile(aes(fill =rescale),colour = "white") +

#scale_fill_distiller(palette="Spectral",name="value") +

#scale_fill_gradientn(values = seq(0,1,0.2),colours = c('cyan','blue','green','orange','red'),name = "value")+

#scale_fill_viridis(option = "A",name = "value")+

scale_fill_viridis(option = "B",name = "value")+

#scale_fill_viridis_c(name = "value")+

coord_fixed(ratio=1)+

scale_x_continuous(expand = c(0,0))+#这个可以去掉与X轴间隙

scale_x_continuous(expand=c(0,0),

breaks=seq(1975, 2023, by=1)) +

theme_bw()+

theme(axis.text.x = element_text(angle=90,vjust = 0.5, hjust = 0.5))+

theme(axis.text= element_text(size = 12,family="serif"))+

theme(axis.title=element_text(size=14,face="bold"))+

labs(x = "Year", y = "Authors", title = "Authors Distribution Over Time")+

theme(plot.title = element_text(size = 15,hjust = 0.5,face="bold"))+

theme(legend.key.width=unit(3,'mm'),legend.key.height=unit(3,'cm'))+

theme(legend.title = element_text(size = 10,family="serif"))+

theme(panel.grid.major =element_blank(),

panel.grid.minor = element_blank(),

panel.background = element_blank(),#去除背景

panel.border = element_blank())#去除边框

## Yearly occurrences of the top 30 journals

Yearly occurrences of the top 30 journals

setwd("D:\\桌面\\肿瘤浸润淋巴细胞\\R分析结果")

library(reshape2)

library(tidyverse)

library(bibliometrix)

#数据读入法

Bw<-read.csv("Bibliometrix-Export-File-2024-05-10.CSV")#biblioshiny获取

topKW=KeywordGrowth(Bw, Tag = "SO", sep = ";", top=30, cdf=FALSE)

topKW2=melt(topKW, id='Year')

#折线图

ggplot(topKW2,aes(Year,value, group=variable, color=variable))+geom_line()+theme_bw()

#标准化

library(plyr)

library(scales)

data <- ddply(topKW2, .(variable),transform,rescale = rescale(value))

#rescale()函数内默认参数to = c(0,1)，所以标准化范围也可以自定义

#热图

library("viridis")#渐变色

ggplot(data, aes(Year, variable)) +

geom_tile(aes(fill =rescale),colour = "white") +

#scale_fill_distiller(palette="Spectral",name="value") +

#scale_fill_gradientn(values = seq(0,1,0.2),colours = c('cyan','blue','green','orange','red'),name = "value")+

#scale_fill_viridis(option = "A",name = "value")+

scale_fill_viridis(option = "B",name = "value")+

#scale_fill_viridis_c(name = "value")+

coord_fixed(ratio=1)+

scale_x_continuous(expand = c(0,0))+#这个可以去掉与X轴间隙

scale_x_continuous(expand=c(0,0),

breaks=seq(1975, 2023, by=1)) +

theme_bw()+

theme(axis.text.x = element_text(angle=90,vjust = 0.5, hjust = 0.5))+

theme(axis.text= element_text(size = 8,family="serif"))+

theme(axis.title=element_text(size=14,face="bold"))+

labs(x = "Year", y = "Journals", title = "Journals Distribution Over Time")+

theme(plot.title = element_text(size = 15,hjust = 0.5,face="bold"))+

theme(legend.key.width=unit(1.4,'mm'),legend.key.height=unit(1.4,'cm'))+

theme(legend.title = element_text(size = 10,family="serif"))+

theme(panel.grid.major =element_blank(),

panel.grid.minor = element_blank(),

panel.background = element_blank(),#去除背景

panel.border = element_blank())#去除边框
